# Impact of Spiritual Support Interventions on the Quality of Life of Patients Who Receive Palliative Care: A Systematic Review

**DOI:** 10.3390/nursrep14030142

**Published:** 2024-08-02

**Authors:** Virginia Prieto-Crespo, Pedro Arevalo-Buitrago, Estefanía Olivares-Luque, Aurora García-Arcos, Pablo Jesús López-Soto

**Affiliations:** 1 Toledo University Hospital, Avda. Rio Guadiana, 45007 Toledo, Spain; n92prcrv@uco.es; 2 Critical Care Service, Reina Sofia University Hospital, Avda. Menendez Pidal s/n, 14004 Córdoba, Spain; aurora.garcia.arcos.sspa@juntadeandalucia.es; 3 Department of Nursing, Pharmacology and Physiotherapy, Cordoba University, Avda Menendez Pidal s/n, 14071 Córdoba, Spain; n82losop@uco.es; 4 Maimonides Biomedical Research Institute of Cordoba, Avda. Menendez Pidal s/n, 14004 Córdoba, Spain; 5 Montilla Hospital, Km. 65,350 Crta. Montoro-Puente Genil A-309, 14550 Montilla, Spain; estefania.olivares.sspa@juntadeandalucia.es

**Keywords:** counselling, palliative care, quality of life, spirituality

## Abstract

Background: Palliative care focuses on the prevention of worsening health, improving the quality of the patient’s life, and the relief of suffering, and therefore has a considerable impact on both the patient suffering from a life-threatening or potentially life-threatening illness and on their family. Spirituality, as the dimension of human life involving the search for meaning, purpose, and transcendence, and connection with oneself, others, and the sacred, could be essential in supporting these patients. The aim of this study was to synthesise the scientific evidence describing the interventions and/or activities undertaken to meet the spiritual needs of the palliative patient. Methods: A literature search was carried out across the following databases: PubMed, LILACS, Scopus, and Web of Science. The PRISMA statement was used to guide this review. Results: Twenty-four articles were included. The thematic categories included spiritual needs at the end of life, the influence of music and dance as palliative care, care for family caregivers, and the comparison between counselling and dignity therapy. Conclusions: Interventions in the biopsychosocial–spiritual spheres impact on the patient’s peace of mind and promote the acceptance of a “good death”. Healthcare personnel play an essential role in the way their patients prepare for the moment of death, and the meaning and values they convey help them to accompany and welcome patients. Last but not least, universities can play a crucial role by training nurses to integrate spiritual interventions such as music and dance, or by considering the family as a unit of care. The systematic review protocol was registered in the Prospective International Register of Systematic Reviews (PROSPERO) under protocol number CRD42023490852.

## 1. Introduction

Palliative care is focused on the prevention of worsening symptoms, improving the patient’s quality of life and, consequently, alleviating the suffering of those patients with a life-threatening or potentially life-threatening illness [1,2]. In this process, it is important to emphasise the importance of family care, as the family is the fundamental nucleus of support for the patient, and patient and family together should be treated as a unit, through early identification, assessment and the correct treatment [3,4,5].

The symptoms of palliative patients vary, depending on the nature and the stage of the illness, with assessment of the patient’s spiritual well-being being a critical and fundamental aspect of holistic and multidisciplinary care [6,7,8,9,10].

The Spirituality Guide (*Guía de Espiritualidad*) produced by the Spanish Society of Palliative Care defines spirituality as “the dynamic dimension of human life that relates to the way in which the person (individual or community) experiences, expresses and/or looks for meaning, purpose and transcendence, and the way in which they connect with the moment, with themselves, with others, with nature, with the significant and/or with the sacred” [11]. Indeed, in recent decades, spirituality has been recognised by researchers as an important resource for coping with the distress that accompanies terminal illness [12].

According to the WHO, counselling is defined as a dynamic process in which two people help each other to gain mutual understanding. Two key characters are involved in this process: the “helped” as the person suffering the illness and the “helper” as the person in charge of accompanying and guiding the patient to help them resolve their conflicts [13,14]. Counselling is a democratic process in which the patient is accompanied and released, through therapeutic communication, from the loneliness and isolation that palliative illness brings [15,16,17].

Healthcare personnel play an essential role in the way their patients prepare for the moment of death, thus humanizing end-of-life care. In this context, inquiring into the interventions and/or activities carried out by healthcare professionals in the spiritual sphere with patients receiving palliative care may be useful for improving the training of future professionals. 

The main objective of this study was to systematically review and synthesise the scientific evidence on the impact of spiritual support interventions, including music therapy, dance therapy, counselling, and dignity therapy, on the quality of life and spiritual well-being of adult patients in palliative care settings.

## 2. Materials and Methods

### 2.1. Study Design

A systematic review which is a rigorous method of synthesizing research evidence by systematically collecting, appraising, and analysing studies that meet pre-defined criteria was carried out and presented according to the criteria established by the PRISMA statement (Preferred Reporting Items for Systematic reviews and Meta-Analyses) [18]. The systematic review protocol was registered in the Prospective International Register of Systematic Reviews (PROSPERO) under protocol number CRD42023490852. PROSPERO is an international database for prospectively registering systematic reviews. Registering our review in PROSPERO helps maintain transparency in the review process and allows others to access our protocol to understand the planned methods and objectives.

### 2.2. Study Selection and Data Extraction

A thorough search of the literature was conducted between November and December 2023 and updated in July 2024 using four databases: PubMed, Scopus, LILACS and Web of Science (WOS). To find the articles, we used a search strategy based on terms related to the spiritual approach, palliative care and quality of life (Table 1). The screening procedures were carried out independently by two reviewers (VPC and PAB) using the Rayyan platform. In the case of conflicting opinions over the inclusion or non-inclusion of an article, a third reviewer (PJLS) was consulted.

The following data for each study were extracted: authors’ names, year of publication, country, period of study, sample size, study design, types of intervention tested, and main results.

### 2.3. Criteria for Inclusion

Prior to starting the research, the inclusion and exclusion criteria were established. We included articles in which (i) the study design was experimental or quasi-experimental; (ii) the study subjects were adults over 18 years of age receiving palliative care; (iii) counselling and/or spiritual counselling were carried out. Counselling is understood as “giving advice or assistance to individuals with educational or personal problems”; while spiritual counselling is understood as “reflecting on the fundamental condition of a person’s sense of self to provide a holistic view of the care received” [19]; (iv) the impact of the intervention on the quality of life of the palliative patient and their family was analysed; (v) the language of publication was Spanish or English; (vi) the publication dates ranged from 1 January 2012 to July 2024.

In contrast, articles were excluded if (i) the study design was observational, qualitative, or any other design which was not experimental or quasi-experimental; (ii) the participants were receiving care (but not palliative care); (iii) the research concerned other situations in which the inclusion criteria were not met: participants under 18 years of age; language of publication not Spanish or English; publication date prior to 2012.

### 2.4. Quality Assessment and Risk of Bias

The NIH (National Heart, Lung and Blood Institute) “Study Quality Assessment Tools” checklists was used to assess the quality of the evidence included in the studies, which also depended on the design [20]. The risk of bias was assessed using the Cochrane risk-of-bias assessment tool for randomised trials (RoB 2) [21].

### 2.5. Data Synthesis

In synthesizing the results, we employed a thematic categorical approach. This method was chosen due to the considerable heterogeneity of the studies selected for our review. The studies varied widely in terms of their design, interventions, outcomes measured, and patient populations. Consequently, a thematic analysis allowed us to systematically categorise and analyse the findings across different dimensions. By identifying common themes and patterns, we were able to integrate diverse pieces of evidence and draw meaningful conclusions about the impact of spiritual support interventions on the quality of life and spiritual well-being of palliative care patients.

## 3. Results

### 3.1. Study Selection

**Figure 1:**
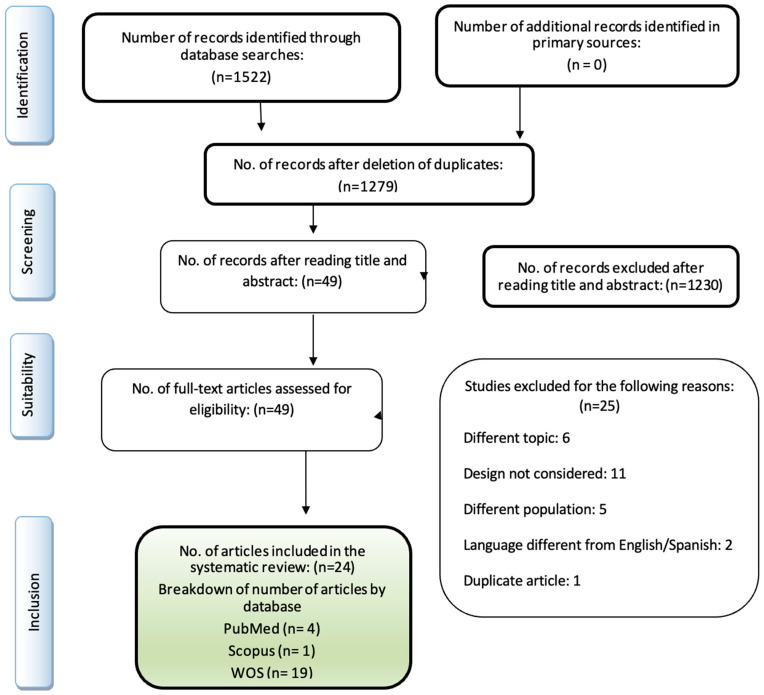
Study selection process (PRISMA) flowchart.

A total of 1522 articles were identified from the initial search of databases. After removing duplicates *(n* = 243), the title and abstract of 1279 records were assessed, leaving a selection of 49 selected at this stage. No additional references were identified through a manual search in websites or reference lists. After a full reading of the 49 articles, 25 of them were excluded: 6 for having a different topic, 11 articles for being a different type of research study, 5 for being conducted in a non-palliative population, 2 for being written in a language other than English/Spanish and 1 article for being duplicated. Finally, 24 articles were included in the systematic review (Figure 1).

### 3.2. Study Characteristics

The studies addressed diverse outcome variables, including quality of life, spiritual well-being, emotional functioning, and physical health indicators. The interventions tested also varied widely, encompassing counselling, spiritual counselling, music therapy, dance therapy, dignity therapy, and combinations thereof. The settings and samples were equally diverse, ranging from patient-focused interventions to those that included family caregivers as part of the unit of care.

Regarding the study design of the 24 articles included in this systematic review, seven (29%) were quasi-experimental studies, two (8%) were mixed methods studies and fifteen (63%) were randomised clinical trials. 

The studies were mainly conducted in the USA (25%, n = 6), followed by the Netherlands, Canada, Spain, Kenya and Germany (8%, n = 2, each country) and the rest from China, Australia, Iran, Czech Republic, Belgium, Malaysia and Hong Kong with one article published. The sample sizes of these studies ranged from 10 to 903. 

### 3.3. Risk of Bias and Quality Assesment

**Figure 2:**
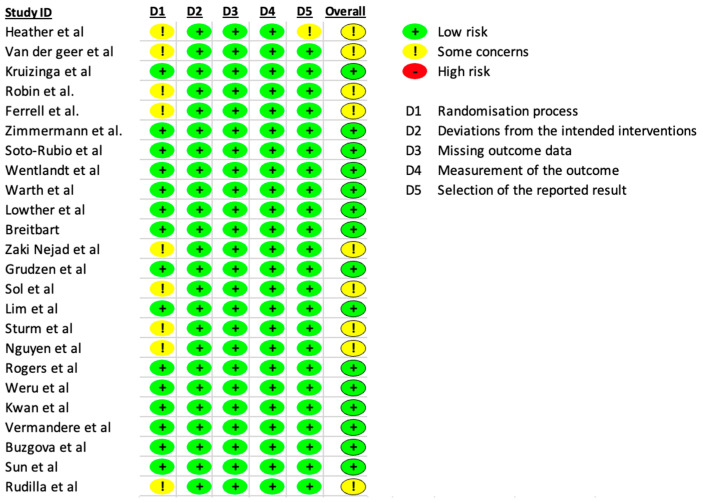
Risk of bias assessment (RoB2) [22,23,24,25,26,27,28,29,30,31,32,33,34,35,36,37,38,39,40,41,42,43,44,45].

Of the 24 studies included, 21 were rated as good after completion of the NIH quality assessment tool, on the other hand, 3 articles rated as fair (Check Supplementary Materials for further information). Among the studies included, thirteen were classified as having a low risk of bias. Nine were classified as having some concerns because they presented deviation from the intended interventions. Figure 2 shows the risk of bias (RoB2) assessment of the included studies.

### 3.4. Data Extraction

After analysing these 24 articles and extracting the data (Table 2), four main approaches were identified:

1. **Benefits of Palliative Care for Spiritual Needs at the End of Life**: This approach explores how palliative care can address spiritual needs, providing patients with meaning and coherence in their lives. It focuses on improving symptoms such as anxiety and depression, which in turn enhances spiritual well-being.
2. **Influence of Dance and Music in Palliative Care**: This approach examines the impact of dance and music therapy on palliative care patients. The studies showed improvements in emotional, social, and physical functioning, as well as spiritual well-being, through interventions like dance classes and music therapy.
3. **Effect of Palliative Care on Family Caregivers**: This approach considers the broader unit of care, including family caregivers. It highlights how palliative care interventions can stabilise anxiety and depression among caregivers and improve their quality of life and spiritual well-being.
4. **Comparison between Counselling and Dignity Therapy**: The fourth approach compares the effectiveness of counselling and dignity therapy in palliative care settings. Both interventions were found to be beneficial, improving quality of life, reducing distress and anxiety, and helping patients maintain a sense of dignity.

These approaches collectively emphasise the holistic nature of palliative care, integrating physical, emotional, and spiritual dimensions to enhance the quality of life for both patients and their caregivers.

#### 3.4.1. Approach 1: Benefits of Palliative Care for Spiritual Needs at the End of Life

At the end of life, new questions about needs arise, but by providing palliative care, healthcare providers can give the patient a great deal of help in finding meaning and coherence in their life [22]. In this context, the quasi-experimental study by Van de Geer et al. reinforces the benefits it accrues for spiritual needs (*p* = 0.008), as well as those for more restful sleep (*p* = 0.020) [23].

Palliative care addresses end-of-life anxiety and depression in patients. Based on this premise, clinical trials by Rogers et al. and Sun et al. demonstrated an improvement in depression and anxiety, which in turn led to spiritual improvement [24,25]. However, several studies found no significant differences [26,27].

Last but not least, Wentlandt et al., in a randomised clinical trial, showed that to facilitate a good death and for the patient to be prepared for it, it is necessary to have good doctor–patient communication, be older, live alone, have good spiritual health and suffer fewer symptoms [28,29,30].

#### 3.4.2. Approach 2: The Influence of Dance and Music in Palliative Care

A randomised clinical trial by Sturm et al. explains the influence of dance on fatigue and quality of life in palliative care patients [31]. Specifically, a sample of 40 patients was studied, in which the intervention group was given dance classes and advice for 5 weeks, unlike the control group, which was only given advice. For the former, this resulted in a significant improvement in fatigue, emotional and social functioning and physical performance (*p* < 0.05) [31].

Finally, music also plays a role in improving palliative care. A randomised clinical trial by Warth et al. with a sample of 104 patients demonstrated the importance of music therapy (“Song of Life”) in palliative care [32]. Specifically, the authors divided the population into two groups: one was given music therapy in addition to the usual care (52 patients), while the other (52 patients) was given a relaxation intervention plus the usual care. The results showed differences with respect to spiritual well-being (*p* = 0.04) and ego integrity (*p* < 0.01), which were further improved after music therapy. Furthermore, momentary distress was significantly lower after music therapy (*p* = 0.05), thus enhancing the patients’ well-being [32].

#### 3.4.3. Approach 3: Effect of Palliative Care on Family Caregivers

The “unit to be addressed” in the spiritual approach is made up of the patient and their family caregivers. Not only can palliative care bring a significant improvement for the patient, but there are also further benefits for the caregiver. In the present review, three studies addressed this theory, demonstrating the benefits of palliative care for primary caregivers.

Hearther R et al. carried out a quasi-experimental study, dividing the study population into two groups: one underwent the LifeCourse intervention (motivational interviewing, individualised person-centred care at the end of life and home visits), while the other group were given the usual care. The LifeCourse intervention stabilised anxiety and depression among family caregivers, while in the other group, anxiety and depression increased [33].

A quasi-experimental study conducted by Nguyen et al. also demonstrated the benefit of palliative care for caregivers, showing an improvement for caregivers in three domains: quality of life (*p* = 0.05), spiritual well-being (*p* = 0.03) and care preparation (*p* = 0,04) [34]. Another study demonstrating the benefits for caregivers was the randomised clinical trial by Kozáková et al. involving 291 participants [35].

#### 3.4.4. Approach 4: Comparison between Counselling and Dignity Therapy for Palliative Patients

Dignity therapy is, by definition, “unique and individualised psychotherapy that enables the patient to review their lives and find meaningful events, people and experiences, thus maintaining their dignity”. This is a key factor in alleviating suffering at the time of death and in fostering hope, self-esteem and meaning in life [36,37].

Two of the studies selected in the present review focus on the search for the benefits of dignity therapy, resulting in an improvement in quality of life in the intervention group (*p* = 0.001), together with improvements in nausea and vomiting (*p* = 0.02), appetite (*p* = 0.02), insomnia (*p* < 0.01) and constipation (*p* = 0.001), as well as an improvement in physical and emotional functioning [36,37,38].

A quasi-experimental study with a sample of 30 patients, carried out by Rudilla et al., shows a comparison of the efficacy of the counselling technique with dignity therapy in palliative patients. The results show that both techniques are equally beneficial as they increase quality of life, and reduce distress and anxiety, the latter being improved more by the counselling technique [37].

## 4. Discussion

This review reflects the importance of a spiritual approach to palliative patients and of meeting their needs at the end of life in order to provide holistic and quality care.

Rego et al. (2020) demonstrated through their cross-sectional study that spiritual well-being increased the patient’s quality of life and that this was associated with greater independence, increased optimism and self-esteem, thus leading to a decrease in depression [46]. Interestingly, the same authors refer to the lack of training of the healthcare staff themselves. In fact, while most of the participating patients emphasised the importance of meeting spiritual needs at the end of life, it was shown that for the majority of them, these needs were not met. This could be put down, in part, to a lack of training: as the health workers were not trained or prepared to act accordingly, they simply did not intervene, because of the unease they experienced when dealing with these situations [46,47,48].

The value of dance and music as an intervention in palliative care has also been highlighted in this review [31,32]. In particular, the study by Woolf et al. states that dance, together with gentle movement, allows the patient to channel interconnected physical and emotional pain. In doing so, it seeks to release the user’s tension and anxiety by allowing them to express themselves as a whole person [49].

Another study concurred that music relieves physical, emotional and spiritual distress and is also useful for improving relaxation and sleep when combined with movement and deep breathing. Thanks to music, positive feelings flourish, leading to a more satisfactory relationship between the palliative patient and their family caregiver. However, the lack of knowledge and limited resources among nurses is an obstacle to these interventions, because, while the nurses are fully aware of the benefits, they often do not know how to use or implement the techniques. Again, this affects not only the patient, but also the primary caregiver [50].

Other interventions that meet patients’ spiritual needs include counselling and dignity therapy. In these areas, the findings, confirmed by other authors [51,52] show that there is a component of therapeutic counselling embedded in dignity therapy; as the techniques are similar, they produce similar results [51].

This systematic review has several limitations. Firstly, the search strategy was carried out using four different databases and so, although it is true that they contain a large number of references, we cannot rule out the possibility that some studies were left out which did not feature in these databases. Secondly, regarding the article selection process, the two main screening reviewers had limited experience, although they were guided and advised by experienced researchers. In utilizing AI and the Rayyan platform for screening articles, we recognise several limitations that were addressed to ensure the rigour of our review. AI tools can introduce biases based on the training data and algorithms used. To mitigate this, we employed a combination of automated and manual screening processes. Two independent reviewers (VPC and PAB) conducted the initial screening using Rayyan, followed by a manual review to verify the AI’s selections. Any discrepancies were resolved by consulting a third reviewer (PJLS). This hybrid approach allowed us to benefit from the efficiency of AI while maintaining the accuracy and reliability of human judgment. Finally, while “counselling” was included as a spiritual intervention, the lack of specific differentiation between general counselling and spiritual counselling leaves some findings incomplete and unfocused. Counselling in this context was broadly defined as providing advice or assistance, which may not fully encapsulate the spiritual aspects intended to be studied. This limitation should be acknowledged as it may have led to an overgeneralisation of the results regarding spiritual interventions. Future studies should aim to more clearly distinguish between general counselling and spiritual counselling to better focus on the spiritual dimensions of patient care.

Based on the findings of our review, particularly the improvements in spiritual well-being and quality of life as observed in the studies by Warth et al. (2021) [32], it is clear that universities can play a crucial role by training nurses to integrate spiritual interventions such as music and dance, or by considering the family as a unit of care. These insights form the basis for rethinking approaches in palliative care, ultimately helping patients to experience a good death, free from suffering.

## 5. Conclusions

This systematic review highlights the manifold benefits of nursing interventions for the spiritual well-being of palliative patients, emphasizing the importance of biopsychosocial–spiritual care. Our findings suggest that integrating spiritual support, such as music and dance, can significantly enhance the quality of life for both patients and their families, fostering positivity, intimacy, and meaningful memories before the final farewell.

The limited use of spiritual interventions in clinical practice underscores the need for improved training and awareness among healthcare professionals. By reflecting on the importance of addressing spiritual needs, this study aims to inspire better quality palliative care that holistically supports patients and their families. Educational institutions can play a crucial role in this by incorporating training on spiritual interventions into their curricula, thus preparing future nurses to deliver comprehensive care.

Additionally, the similarities between counselling and dignity therapy, both of which effectively address spiritual needs and alleviate distress, highlight the potential for these therapies to be integrated into standard palliative care practices. Further research could explore the long-term benefits of these interventions, their cost-effectiveness, and the development of standardised protocols to ensure consistent implementation.

In summary, this study not only sheds light on the significant impact of spiritual support on palliative care but also calls for continued research and education to enhance the holistic care of patients facing life-threatening illnesses.

## Figures and Tables

**Table 1:** Search strategies used in the three databases.

Database	Search Strategy
**Pubmed**	*(“Counseling” [Mesh] OR “counseling”[tiab] OR “directive counseling”[tiab] OR “motivational interviewing”[tiab] OR “Distance counseling”[tiab] OR “pastoral care” [tiab] OR “sex counseling”[tiab] OR “spiritual care”[tiab] OR “spiritual counseling”[tiab]) AND (“terminal care”[Mesh] OR “palliative care”[Mesh] OR “hospice care”[Mesh] OR “terminal care”[tiab] OR “care, terminal”[tiab] OR “end of life care” [tiab] OR “end-of-life care” [tiab] OR “care, end-of-life”[tiab] OR “end-of-life cares”[tiab] OR “palliative care”[tiab] OR “care, palliative”[tiab] OR “palliative treatments”[tiab] OR “palliative treatment”[tiab] OR “treatment, palliative”[tiab] OR “treatments, palliative”[tiab] OR “therapy, palliative”[tiab] OR “palliative therapy”[tiab] OR “palliative supportive care”[tiab] OR “supportive care, palliative”[tiab] OR “palliative surgery”[tiab] OR “surgery, palliative”[tiab] OR “hospice care”[tiab] OR “care, hospice”[tiab] OR “hospice programs”[tiab] OR “hospice program”[tiab] OR “program, hospice”[tiab] OR “programs, hospice”[tiab] OR “bereavement care”[tiab] OR “care, bereavement”[tiab]) AND (“Quality of Life”[Mesh] OR “QoL”[tiab] OR “HRQOL” [tiab] OR “quality of life” [tiab] OR “life quality” [tiab])*
**Scopus**	*TITLE-ABS(“Counseling” OR “directive counseling” OR “motivational interviewing” OR “Distance counseling” OR “pastoral care” OR “sex counseling” OR “spiritual care” OR “spiritual counseling”) AND TITLE-ABS(“terminal care” OR “palliative care” OR “hospice care” OR “care, terminal” OR “end of life care” OR “end-of-life care” OR “care, end-of-life” OR “end-of-life cares” OR “care, palliative” OR “palliative treatments” OR “palliative treatment” OR “treatment, palliative” OR “treatments, palliative” OR “therapy, palliative” OR “palliative therapy” OR “palliative supportive care” OR “supportive care, palliative” OR “palliative surgery” OR “surgery, palliative” OR “care, hospice” OR “hospice programs” OR “hospice program” OR “program, hospice” OR “programs, hospice” OR “bereavement care” OR “care, bereavement”) AND TITLE-ABS (“Quality of Life” OR “QoL” OR “HRQOL” OR “quality of life” OR “life quality”)*
**Web of Science**	*(AB=(Counseling) OR AB=(directive counseling) OR AB=(motivational interviewing) OR AB=(Distance counseling) OR AB=(pastoral care) OR AB=(sex counseling) OR AB=(spiritual care) OR AB=(spiritual counseling)) AND (AB=(terminal care) OR AB=(hospice care) OR AB=(palliative care) OR AB=(care, terminal) OR AB=(end of life care) OR AB=(end-of-life care) OR AB=(care, end-of-life) OR AB=(end-of-life cares) OR AB=(care, palliative) OR AB=(palliative treatments) OR AB=(palliative treatment) OR AB=(treatment, palliative) OR AB=(treatments, palliative) OR AB=(therapy, palliative) OR AB=(palliative therapy) OR AB=(palliative supportive care) OR AB=(supportive care, palliative) OR AB=(palliative surgery) OR AB=(surgery, palliative) OR AB=(care, hospice) OR AB=(hospice programs) OR AB=(hospice program) OR AB=(program, hospice) OR AB=(programs, hospice) OR AB=(bereavement care) OR AB=(care, bereavement)) AND (AB=(quality of life) OR AB=(QoL) OR AB=(HRQOL) OR AB=(life quality))*
**LILACS**	*(“Counseling” OR “directive counseling” OR “motivational interviewing” OR “Distance counseling” OR “pastoral care” OR “sex counseling” OR “spiritual care” OR “spiritual counseling”) AND (“terminal care” OR “palliative care” OR “hospice care” OR “care, terminal” OR “end of life care” OR “end-of-life care” OR “care, end-of-life” OR “end-of-life cares” OR “care, palliative” OR “palliative treatments” OR “palliative treatment” OR “treatment, palliative” OR “treatments, palliative” OR “therapy, palliative” OR “palliative therapy” OR “palliative supportive care” OR “supportive care, palliative” OR “palliative surgery” OR “surgery, palliative” OR “care, hospice” OR “hospice programs” OR “hospice program” OR “program, hospice” OR “programs, hospice” OR “bereavement care” OR “care, bereavement”) AND (“Quality of Life” OR “QoL” OR “HRQOL” OR “quality of life” OR “life quality”) AND (db:(“LILACS”))*

**Table 2:** Summary of included studies.

Author (Year)	Country	Period	Sample	Design	Intervention vs. Comparator	Trigger Variable	(NIH Quality Tool)
Soto-Rubio A, et al. (2020) [22]	Spain	Not provided	60 patients of legal age with advanced or terminal illness and who are cognitively well.	Randomised controlled trial	Patients with standard care versus those provided with the **Kibo interview.**	Transpersonal spirituality and resilience improved, and demoralisation decreased. The opposite happened in the control group.	12/14
Van der Geer J, et al. (2016) [23]	Netherlands	13 months	85 patients receiving palliative care with a life expectancy of more than 12 months.	Quasi-experimental study	**Training provided by chaplains** to health personnel. Indicators are observed 1 month after and before the intervention.	There is a significant effect (*p* = 0.008) in the health professionals’ attention to patients’ spiritual and existential needs and in favour of patients’ sleep (*p* = 0.020).	10/12
Rogers J, et al. (2017) [24]	United States	6 months	150 patients with advanced heart failure and a high risk of 6-month mortality.	Prospective clinical trial	Usual care or usual care+ **multidimensional palliative care intervention** (UC+PAL))	Depression improved in UC+PAL patients (*p* = 0.020), with similar results for anxiety (*p* = 0.048). Spiritual well-being also improved.	10/14
Sun XH, et al. (2021) [25]	China	Not provided	100 patients over 18 years of age with a histological or cytological diagnosis of a stage III or IV malignant tumour with tumour node metastasis.	Randomised cluster clinical trial.	Current routine care (control group) versus **advanced cancer spiritual care intervention** in addition to routine care (experimental group)	The overall spiritual health score of the experimental group was higher. The proportion of patients without anxiety was significantly higher (95.45% vs. 60.98%). The proportion of non-depressed patients and quality of life was also higher.	11/14
Grudzen CR, et al. (2016) [26]	United States	12 weeks	136 patients with known advanced cancer admitted to or observed in hospital.	Single-blind randomised clinical trial	**ED-initiated palliative care consultation for advanced cancer** patients versus usual care.	Quality of life was higher in the intervention group. Survival estimates were longer, although there was no statistical significance. There were also no differences in depression, ICU admission and discharge to hospice.	13/14
Kwam C, et al. (2019) [27]	Hong Kong	Not provided	109 patients of legal age with a life expectancy of not less than one month	Mixed methods study (randomised controlled trial and qualitative evaluation)	Usual care versus **short-term life review intervention**	The intervention group showed an improvement in spiritual well-being. Depression and anxiety also improved, although not significantly.	13/14
Kruizinga R, et al. (2019) [28]	Netherlands	2014–2016 (20 months)	153 patients over 18 years of age with a life expectancy of more than 6 months.	Randomised controlled trial	Usual care versus intervention with a **spiritual advisor**	There are no significant changes in quality of life and well-being between groups. Quality of life was associated with peace (β = 0.52) and life satisfaction (β = 0.61).	11/14
Wentlandt K, et al. (2012) [29]	Canada	4 months	469 patients with stage IV gastrointestinal, genitourinary, breast or gynaecological cancer or stage III/IV lung cancer; and a clinical prognosis of 6 months to 2 years.	Randomised cluster clinical trial	**Early intervention of the palliative care team** versus routine cancer care.	31% report worrying about their family members, 27% feel a burden. 20% reported financial stress and 16% were afraid of dying. Better preparation at the end of life was associated with better doctor–patient communication; there were also associations with older age of the patient, living alone.	13/14
Sun V, et al. (2015) [30]	United States	12 weeks	475 non-small cell lung cancer patients scheduled for treatment and 354 family caregivers	Quasi-experimental prospective study	Usual care versus **palliative care**.	The palliative care group scored best for meaning and peace, and harmony.	11/12
Sturm I, et al. (2014) [31]	Germany	5 weeks	40 patients of legal age and who are fatigued in active cancer treatment	Randomised controlled clinical trial	Advice versus advice and **dance classes**	The intervention group (dance) improved fatigue (36% reduction). Quality of life was also improved: emotional and social functioning scales and physical performance (*p* < 0.05).	8/14
Warth M, et al. (2021) [32]	Germany	Not provided	104 patients receiving palliative treatment, over the age of majority and with a life expectancy of less than 12 months.	Multicentre randomised controlled trial	**Music therapy** and usual care versus relaxation and usual care.	No significant differences in the primary outcome of psychological quality of life. Spiritual well-being was higher in music therapy (*p* = 0.04) and ego integrity (*p* < 0.01), as well as lower distress (*p* = 0.05).	13/14
Britt HR, et al. (2019) [33]	United States	30 months	903 patients receiving palliative care with a life expectancy of more than 3 years	Quasi-experimental intervention study	Patients who have usual care (UC) versus those who have **LifeCourse (LC).**	LC patients show greater improvement in communication and attention than the UC group (*p* = 0.016). Caregivers of UC patients show greater anxiety and depression.	11/12
Nguyen HQ, et al. (2018) [34]	United States	3 months	202 patients of legal age with non-small cell lung cancer and 122 FCG (family caregivers).	Quasi-experimental study	Patients with usual care versus **palliative care**	Patients improved physical, emotional and functional well-being after palliative care (*p* < 0.01). Caregivers improved quality of life (*p* = 0.05), spiritual well-being (*p* = 0.03) and caregiving preparation (*p* = 0.04).	11/12
Kozáková R, et al. (2020) [35]	Czech Republic	3 months	291 participants of legal age (151 with progressive neurological disease and 140 family carers).	Randomised controlled trial study design	Standard care (control group) versus **multidisciplinary palliative team consultations** (intervention group)	Differences in symptom burden (*p* < 0.001), emotional burden (*p* < 0.001), social functioning (*p* = 0.046), spiritual area (non-religious) and quality of life, also in family members.	11/14
Weru J, et al. (2020) [36]	Kenya	6 weeks	126 adults aged 18–65 with advanced cancers.	Randomised control trial	**Dignity therapy** versus usual therapy	The dignity therapy group showed no statistical improvement in quality of life. It did show a trend towards anxiety (*p* = 0.059) and improvement in appetite, reduction in anxiety and improvement in well-being were observed.	12/14
Rudilla D, et al. (2017) [37]	Spain	2 months	30 patients with advanced or terminal illness and who show an interest in dignity therapy.	Quasi-experimental design.	**Dignity therapy versus counselling**	The counselling group improved in distress, quality of life and two of the dignity dimensions (existential and dependency distress). The results of the dignity group were similar, except for anxiety, which did not improve after the intervention. There were no significant differences between the two therapies.	11/12
Zaki-Nejad M, et al. (2020) [38]	Iran	2017–2018	50 patients diagnosed with stage III or IV cancer, who are aware of their disease. Older than 18 years of age and without cognitive impairment or mental illness.	Quasi-experimentalstudy	Patients with usual care versus patients with **dignity therapy**.	Dignity therapy improved quality of life (*p* = 0.001), nausea and vomiting (*p* = 0.02), insomnia (*p* < 0.001), appetite (*p* = 0.02), constipation (*p* < 0.001), physical and emotional functioning.	11/12
Keall RM, et al. (2013) [39]	Australia	Not provided	10 patients with a life-threatening disease, with a life expectancy of less than 2 years.	Mixed methods study (thematic analysis of audiotaped session pre-and post-intervention)	**Nurse-facilitated life preparation** and end-of-life intervention.	8 out of 10 patients found it useful. 7 patients reflected on their life. 9 patients would recommend it.	13/14
Ferrell B, et al. (2015) [40]	Not provided	2011–2014	491 palliative care patients.	Quasi-experimental prospective study	Patients with usual care versus patients with **interdisciplinary and supportive care**	The intervention group improved quality of life (109.1 versus 101.4; *p* < 0.001), symptomatology (25.8 versus 23.9; *p* < 0.001), and spiritual well-being (38.1 versus 36.2; *p* = 0.001). In addition, less psychological distress was found (2.2 versus 3.3; *p* < 0.001).	11/12
Zimmermann C, et al. (2014) [41]	Canada	4 months	461 patients of legal age, with advanced cancer, a European Cooperative Oncology Group performance status of 0 to 2 and a clinical prognosis of 6 to 24 months.	Randomised cluster-controlled trial.	Standard patient care versus early **comprehensive palliative care and multidisciplinary assessment of distress and support.**	The intervention group did not improve quality of life, as measured by the FACIT-Sp scale at 3 months, although it did improve according to the QUAL-E scale. Satisfaction with care also improved. At 4 months, there were significant changes except in CARES-MIS.	12/14
Lowther K, et al. (2015) [42]	Kenya	4 months	120 patients taking antiretroviral drugs with pain	Randomised controlled trial	Patients with usual care versus **palliative care patients.**	The intervention had no significant effect on pain (*p* = 0.95). However, there was an improvement in the intervention group for the mental health dimension.	11/14
Lim MA, et al. (2021) [43]	Malaysia	2 months	60 patients of legal age in palliative care, with overall distress score ≥ 4/10 (according to distress pictogram).	Randomised controlled trial	**Regular meditation (control) versus 5-min love mindfulness (intervention)**	Significant improvements in overall and total distress score and spiritual quality of life. Worry, anger, non-acceptance and emptiness also improved.	13/14
Vermandere M, et al. (2015) [44]	Belgium	6 weeks	99 patients with progressive, potentially life-threatening disease.	Randomised controlled trial	Usual care versus **structured spiritual history.** Both of them at home.	There was no significant change. No demonstrable effect on SWB, quality of life, patient-provider trust or pain.	8/14
Breitbart W, et al. (2018) [45]	United States	4 months	321 patients with stage IV solid tumour cancer and at least moderate distress and who are of legal age.	Randomised controlled trial	**Patients with individual meaning centred psychotherapy (IMCP) versus those with supportive psychotherapy (SP)** versus enhanced usual care	The effect of IMCP was significantly larger than the effect of SP for quality of life and sense of meaning, but not for the rest of the variables. IMCP would result in significantly greater improvements than the other two.	11/14

## Data Availability

No new data were created or analysed in this study. Data sharing is not applicable in this study.

## Supplementary Materials

The following supporting information can be downloaded at: https://www.mdpi.com/article/10.3390/nursrep14030142/s1, Table S1: Quality assessment of quasi-experimental studies. Table S2: Quality assessment of mixed methods studies. Table S3: Quality assessment of randomised clinical trials.

## Public Involvement Statement

No public involvement in any aspect of this research.

## Guidelines and Standards Statement

This manuscript was drafted following the Preferred Reporting Items for Systematic Reviews and Meta-Analysis (PRISMA) guidelines.
