# Supplementary material for: Impact of Spiritual Support Interventions on the Quality of Life of Patients Who Receive Palliative Care: A Systematic Review
Source: Nurs Rep. 2024 Aug 2;14(3):1906–21. doi: 10.3390/nursrep14030142 (PMC11348222; doi:10.3390/nursrep14030142)
Supplement: Supplementary file 1 [file nursrep-14-00142-s001.zip › nursrep-3095941-supplementary.pdf]

Annexe 1. Quality assessment

Table 1. Quality assessment of quasi-experimental studies

Table 2. Quality assessment of mixed methods studies

Table 3. Quality assessment of randomised clinical trials

Table 1. Quality assessment of quasi-experimental studies

	STUDIES (following the bibliography)						
NIH CRITERIA	33	23	40	38	25	34	37
1. Was the question or objective of the study clearly stated?	✔	✔	✔	✔	✔	✔	✔
2. Were the eligibility/selection criteria for the study population pre-specified and clearly described?	✔	✔	✔	✔	✔	✔	✔
3. Were the study participants representative of those who would be eligible for the test/service/intervention in the general or clinical population of interest?	✔	✔	✔	✔	✔	✔	✔
4. Did all eligible participants who met the pre-specified entry criteria enrol?	✓	✔	✔	✔	✔	✔	✔
5. Was the sample size large enough to provide confidence in the findings?	✔	✔	✔	✔	✔	✔	✔

6. Was the test/service/intervention clearly described and administered uniformly across the study population?	✓	✔	✔	✔	✔	✔	✔
7. Were the outcome measures pre-specified, clearly defined, valid, reliable and consistently assessed across all study participants?	✔	✔	✔	✔	✔	✔	✔
8. Were the people evaluating the results blinded to the participants' exposures/interventions?	**NR**	**NR**	**NR**	**NR**	**NR**	**NR**	✖
9. Were losses to follow-up after baseline 20% or less? Were losses to follow-up taken into account in the analysis?	✔	**NR**	✔	✔	✔	✔	✔
10. Did statistical methods examine changes in outcomes from pre- to post-intervention? Were statistical tests conducted that provided p-values for pre to post changes?	✔	✔	✔	✔	✔	✔	✔

11. Were outcome measures of interest taken several times before the intervention and several times after the intervention (i.e., did they use an interrupted time series design)?	✔	✔	✔	✔	✔	✔	✔
12. If the intervention was implemented at the group level (e.g. a whole hospital, a community, etc.), did the statistical analysis take into account the use of individual-level data to determine group-level effects?	✔	✔	✔	✔	✔	✔	✔

Table 2. Quality assessment of mix methods studies

NIH CRITERIA	STUDIES (following the bibliography)	
	39	27
1. Was the research question or objective clearly stated in this article?	✔	✔
2. Was the study population clearly specified and defined?	✔	✔
3. Was the participation rate of eligible persons at least 50%?	✔	✔
4. Were all subjects selected or recruited from the same or similar populations (including the same time period)? Were inclusion and exclusion criteria for participation in the study pre-specified and uniformly applied to all participants?	✔	✔
5. Was a justification for the sample size, a description of power or estimates of variance and effect provided?	✔	✔
6. For the analyses of this document, were the exposures of interest measured before measuring outcomes?	✓	✔
7. Was the time frame sufficient so that one could reasonably expect to see an association between exposure and outcome, if any?	✔	✔
8. For exposures that may vary in amount or level, did the study examine different levels of exposure in relation to the outcome (e.g., exposure categories or exposure measured as a continuous variable)?	✔	✔

9. Were the exposure measures (independent variables) clearly defined, valid, reliable and consistently implemented across all study participants?	✔	✔
10. Were exposures assessed more than once over time?	✔	✖
11. Were the outcome measures (dependent variables) clearly defined, valid, reliable and consistently implemented across all study participants?	✔	✔
12. Were the outcome assessors blinded to the exposure status of the participants?	**NR**	✔
13. Were losses during the monitoring after the beginning of 20% or less?	✔	✔
14. Were key potential confounding variables measured and statistically adjusted for their impact on the relationship between exposure(s) and outcome(s)?	✔	✔

Table 3. Quality assessment of randomised clinical trials

	STUDIES (following the bibliography)														
NIH CRITERIA	28	41	22	29	32	43	53	26	44	31	24	36	45	35	30
1. Was the study described as randomised, a randomised trial, a randomised clinical trial or an RCT?	✔	✔	✔	✔	✔	✔	✔	✔	✔	**NR**	✔	✔	✔	✔	✔
2. Was the method of randomisation (i.e. use of randomly generated assignment) appropriate?	✔	✔	✔	✔	✔	✔	✔	✔	✔	✖	✔	✔	✔	✔	✔
3. Was the treatment allocation concealed (so that allocations could not be predicted)?	**NR**	✖	✔	✔	✔	✖	✖	✔	✔	✖	✖	✖	✖	**NR**	**NR**
4. Were study participants and providers blinded to treatment group assignment?	**NR**	✔	✔	✔	✔	✖	✖	✖	✔	**NR**	✖	✔	**NR**	**NR**	**NR**
5. Were the people evaluating the results blinded to the participants' group assignments?	**NR**	✖	✖	**NR**	✖	✔	**NR**	✔	**NR**	**NR**	✖	✖	**NR**	**NR**	**NR**

6. Were the groups similar at the start of the study in important characteristics that could affect the results (e.g. demographics, risk factors, comorbid conditions)?	✔	✔	✔	✔	✔	✔	✔	✔	✔	✔	✔	✔	✔	✔	✔
7. Was the overall study dropout rate at the end of the study 20% or less of the number assigned to the treatment?	✔	✔	✔	✔	✔	✔	✔	✔	✔	✔	✖	✔	✖	✔	✔
8. Was the differential dropout rate (between treatment groups) at the end 15 percentage points or less?	✔	✔	✔	✔	✔	✔	✔	✔	✔	✔	✔	✔	✖	✔	✔
9. Was there high adherence to the intervention protocols for each treatment group?	✔	✔	✔	✔	✔	✔	✔	✔	✔	✔	✔	✔	✔	✔	✔
10. Were other interventions avoided or similar across groups (e.g. similar background treatments)?	✔	✔	✔	✔	✔	✔	✔	✔	✔	**NR**	✔	✔	✔	✔	✔

11. Were outcomes assessed using valid and reliable measures, implemented consistently across all study participants?	✔	✔	✔	✔	✔	✔	✔	✔	✔	✔	✔	✔	✔	✔	✔
12. Did the authors report that the sample size was large enough to be able to detect a difference in the main outcome between groups with at least 80% power?	✔	✔	✔	✔	✔	✖	✔	✔	✔	✔	✔	✔	✖	✔	✔
13. Were the reported outcomes or subgroups analysed pre-specified (i.e. identified prior to conducting the analyses)?	✔	✔	✔	✔	✔	✔	✔	✔	✔	✔	✔	✔	✔	✔	✔
14. Were all randomised participants analysed in the group to which they were originally assigned, i.e. did they use an intention-to-treat analysis?	✔	✔	✔	✔	✔	✔	✔	✔	✔	✔	✔	✔	✔	✔	✔
